# Primary Small Cell Carcinoma of the Liver Presenting With Acute Liver Failure

**DOI:** 10.7759/cureus.111447

**Published:** 2026-06-24

**Authors:** Fred Ahmadi, Brian Nohomovich, Neil Al-Saidi, Mark Devore

**Affiliations:** 1 Internal Medicine, Ascension Providence Hospital, Southfield, USA; 2 Gastroenterology, Ascension Providence Hospital, Southfield, USA; 3 Radiology, Ascension Providence Hospital, Southfield, USA

**Keywords:** chemotherapy, hepatology, liver failure, malignancy, small cell carcinoma

## Abstract

Primary small cell carcinoma of the liver is an exceptionally rare malignancy and must be distinguished from metastatic pulmonary small cell carcinoma by excluding a pulmonary primary. We report a 55-year-old man with suspected alcohol-related cirrhosis and diabetes who presented for elective lithotripsy and was found to have jaundice, hepatomegaly, and acute liver dysfunction. Laboratory evaluation demonstrated marked hyperbilirubinemia and transaminitis with normal alpha-fetoprotein. Imaging revealed interval hepatomegaly with diffuse hepatic involvement compared to CT performed three months earlier, which had shown no focal lesions. Liver biopsy demonstrated small cell carcinoma with immunohistochemical positivity for CD56 and synaptophysin and negative TTF-1 staining. Chest radiograph showed no evidence of pulmonary malignancy, supporting a primary hepatic origin. The patient developed progressive liver failure, hypoxic respiratory failure, and shock and died seven days after admission. A review of 31 previously reported cases demonstrates a predominance in middle-aged males, frequent association with cirrhosis or diabetes, common presentation with abdominal pain and jaundice, and typically normal tumor markers. These findings were consistent with those observed in our patient. Reported survivors most often received combined surgical resection and platinum-based chemotherapy. This case highlights the rare yet aggressive course of primary small cell carcinoma of the liver and its potential to cause rapid liver failure.

## Introduction

Extrapulmonary small cell carcinoma is a rare entity distinct from small cell carcinoma of the lung [1], which accounts for the vast majority of small cell carcinomas. When small cell carcinoma involves the liver, it most often represents metastatic disease from a pulmonary primary. Establishing a true hepatic primary requires exclusion of lung involvement. There are very few reports of extrapulmonary small cell carcinoma and even fewer describing primary hepatic small cell carcinoma. The pathogenesis of primary hepatic small cell carcinoma remains poorly understood. Proposed mechanisms include malignant transformation of hepatic neuroendocrine cells, pluripotent stem cells, or neuroendocrine differentiation within preexisting hepatic epithelium [2,3]. Chronic liver disease, hepatitis, cirrhosis, diabetes mellitus, and tobacco exposure have been reported in some cases, although definitive risk factors have not been established. Extrapulmonary small cell carcinoma is characterized by aggressive behavior and a high rate of recurrence [1-3]. Diagnostic evaluation is challenging because imaging findings can overlap with hepatocellular carcinoma, metastatic disease, lymphoma, severe hepatitis, or other diffuse hepatic processes, making histopathologic confirmation essential [4-6].

We report a 55-year-old man who developed acute liver failure and multisystem organ failure within one week of hospitalization, attributed to primary small cell carcinoma of the liver. CT of the abdomen and pelvis performed three months prior to presentation had shown no hepatic lesions. We also reviewed 31 previously published cases of primary hepatic small cell carcinoma. This case is noteworthy, as primary hepatic small cell carcinoma remains exceptionally rare, is difficult to distinguish clinically and radiographically from more common hepatic processes, and may present with rapid hepatic failure. Reporting this case adds to the limited literature on its diagnostic approach and aggressive clinical course.

## Case presentation

In May 2025, a 55-year-old man with suspected alcohol-related cirrhosis, atrial fibrillation on apixaban, Crohn’s disease, chronic obstructive pulmonary disease, gastroesophageal reflux disease, hypertension, recurrent nephrolithiasis, peripheral artery disease on clopidogrel and aspirin, diabetes mellitus, and tobacco use presented for elective outpatient lithotripsy.

In the preoperative area, the patient developed atrial fibrillation with a heart rate in the 130s and was referred to the emergency department for rate control. He reported flank pain radiating to the groin, consistent with nephrolithiasis, which later became more generalized. This pain had been ongoing for the past three weeks but was progressively worsening. He described it as a sharp pain. Physical examination revealed scleral icterus, jaundice, abdominal distension, and hepatosplenomegaly.

He denied fever, chills, weight loss, confusion, headache, dysphagia, odynophagia, constipation, diarrhea, hematemesis, melena, hematochezia, hematuria, or urinary changes. He had no prior history of decompensated cirrhosis.

Past surgical history included cholecystectomy, ventral hernia repair, right total hip arthroplasty, and tonsillectomy. He was allergic to amoxicillin-clavulanate. Home medications included albuterol, cyclobenzaprine, apixaban, dicyclomine, tamsulosin, sitagliptin, atorvastatin, metoprolol, clopidogrel, inhaled corticosteroid/long-acting muscarinic antagonist/long-acting beta₂-adrenergic agonist, mesalamine, and fenofibrate. He smoked one pack per day, had quit alcohol 15 years earlier, and reported daily cannabis use. Family history was notable only for atrial fibrillation in his father and obstructive sleep apnea and hypertension in his mother. There was no known family history of gastrointestinal malignancy.

Admission laboratory findings are summarized in Table 1. The patient demonstrated marked hyperbilirubinemia, transaminitis, and hyperammonemia. The Model for End-Stage Liver Disease (MELD) score was 23, the Child-Pugh class was B, and the Maddrey discriminant function was 22.

**Table 1:** Laboratory findings on admission WBC: white blood cell count, INR: international normalized ratio, PT: prothrombin time, AST: aspartate aminotransferase, ALT: alanine aminotransferase, GGT: gamma-glutamyl transferase, AFP: alpha-fetoprotein

Parameter	Result	Reference range
WBC	17.5 × 10³/µL	4.0-11.0
Hemoglobin	15.6 g/dL	13.5-17.5
Platelets	258 × 10³/µL	150-400
INR	1.3	0.8-1.2
PT	15.6 sec	11-13.5
Sodium	130 mmol/L	135-145
Potassium	3.1 mmol/L	3.5-5.0
Total bilirubin	10.3 mg/dL	0.2-1.2
Direct bilirubin	8.5 mg/dL	<0.3
Alkaline phosphatase	368 U/L	44-147
AST	412 U/L	10-40
ALT	127 U/L	7-56
Ammonia	77 µmol/L	15-45
GGT	1126 U/L	8-61
AFP	3.2 ng/mL	<10

Based on the presenting symptoms, examination, and laboratory abnormalities, the initial differential diagnosis included acute decompensation of chronic liver disease, obstructive hepatobiliary disease, acute viral or autoimmune hepatitis, drug-induced liver injury, or malignancy.

Chest radiograph on admission showed no evidence of an acute process (Figure 1). Recent outpatient imaging included a limited abdominal ultrasound demonstrating hepatomegaly with a lobulated contour consistent with cirrhosis and a non-obstructing 8 mm left renal calculus, and Doppler imaging showing vascular patency. Images were unavailable for the outpatient workup. Esophagogastroduodenoscopy two years earlier showed no varices.

**Figure 1:**
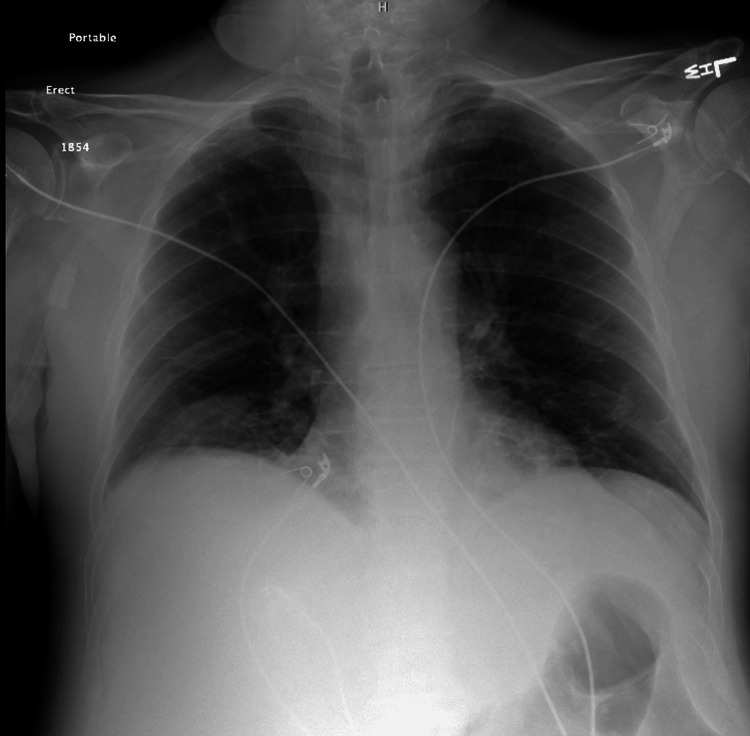
Chest radiograph on admission

Extensive evaluation of alternative causes of liver injury was unrevealing. Viral hepatitis testing, alpha-1 antitrypsin, iron studies, antinuclear antibody, IgG, antimitochondrial antibody, smooth muscle antibody, ceruloplasmin, cytoplasmic antineutrophil cytoplasmic antibody, perinuclear antineutrophil cytoplasmic antibody, liver-kidney microsomal antibody, and total IgG were normal or negative. The serum alpha-fetoprotein level was 3.2 ng/mL. The acetaminophen level was normal. Due to worsening hyperbilirubinemia and coagulopathy, an MRI of the abdomen with and without contrast was obtained (Figure 2).

**Figure 2:**
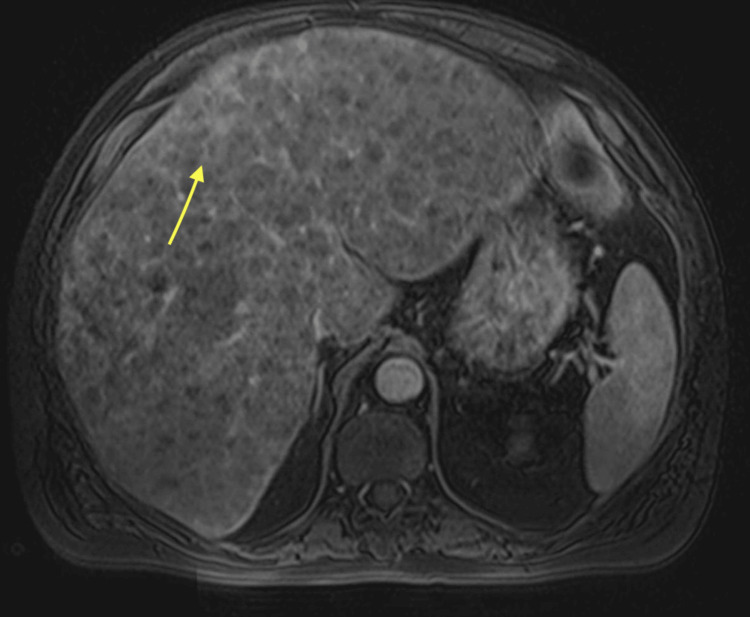
Axial dynamic contrast-enhanced T1-weighted MRI (3D) of the liver demonstrates diffuse infiltrative enhancement with innumerable hypoenhancing foci throughout the hepatic parenchyma consistent with extensive involvement by biopsy-proven primary hepatic small cell carcinoma MRI: magnetic resonance imaging, 3D: three-dimensional

Compared to imaging three months prior, there was marked hepatomegaly. The liver demonstrated a diffuse heterogeneous signal not typical of cirrhosis. Differential considerations included peliosis, diffuse infiltrative disease, or drug-induced hepatitis. A hypo-enhancing subcapsular branching lesion was identified in segments VI and VIII. A 2.2 × 1.8 cm peripheral enhancing lesion in the inferior right lobe with delayed filling was seen (Figure 3).

**Figure 3:**
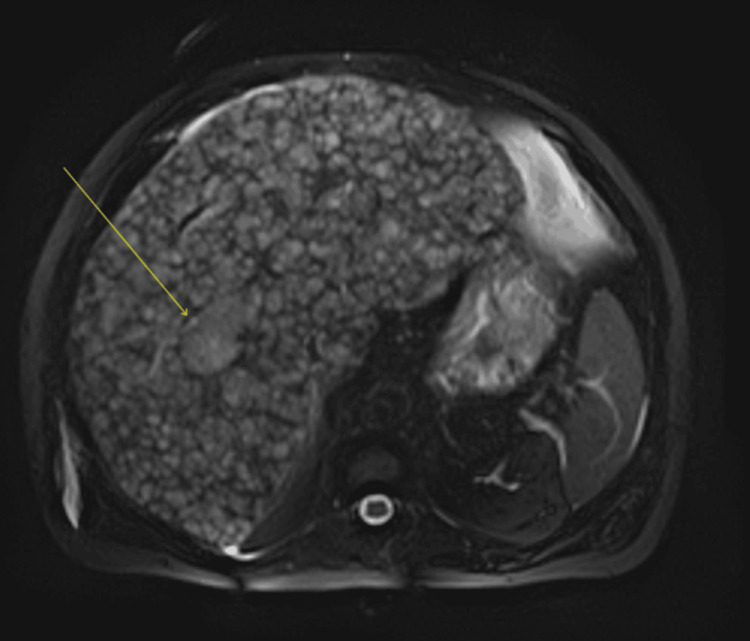
Axial T2-weighted fat-suppressed MRI of the liver (TE = 160 ms) demonstrates a hyperintense hepatic lesion (arrow), corresponding to biopsy-proven primary hepatic small cell carcinoma. Compared with the CT of the abdomen and pelvis performed three months prior, there is interval hepatomegaly with diffuse hepatic parenchymal heterogeneity and widespread infiltrative T2 hyperintensity, consistent with diffuse hepatic involvement MRI: magnetic resonance imaging, CT: computed tomography

Ultrasound-guided liver biopsy demonstrated proliferation of atypical medium-sized cells with irregular hyperchromatic nuclei and scant cytoplasm arranged in nests. Nuclear molding and numerous apoptotic cells were present (Figure 4).

**Figure 4:**
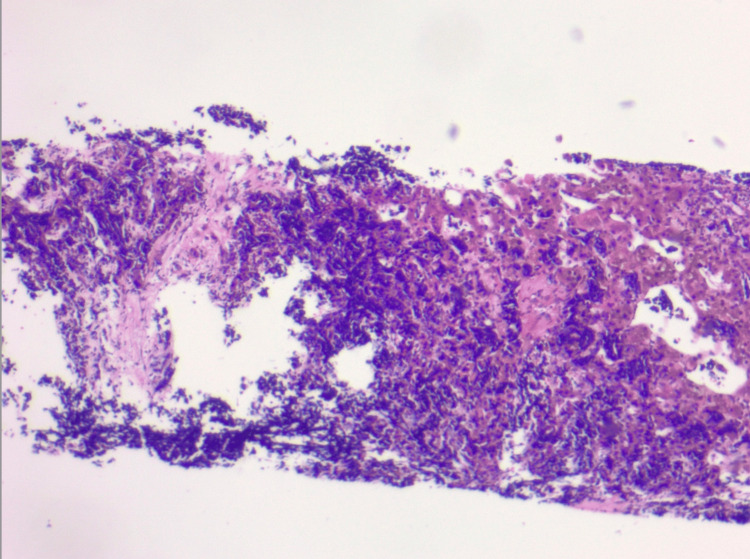
Liver biopsy (40x) showing proliferation of atypical medium-sized cells with irregular hyperchromatic nuclei and scant cytoplasm arranged in nests. Nuclear molding and numerous apoptotic cells are present

Tumor cells were positive for pankeratin (A3), CK7 (A2), CD56 (A3), and synaptophysin (A2) and negative for CD20 (A2), chromogranin (A3), and TTF-1 (A3) (Figures 5-6). These findings established the diagnosis of small cell carcinoma.

**Figure 5:**
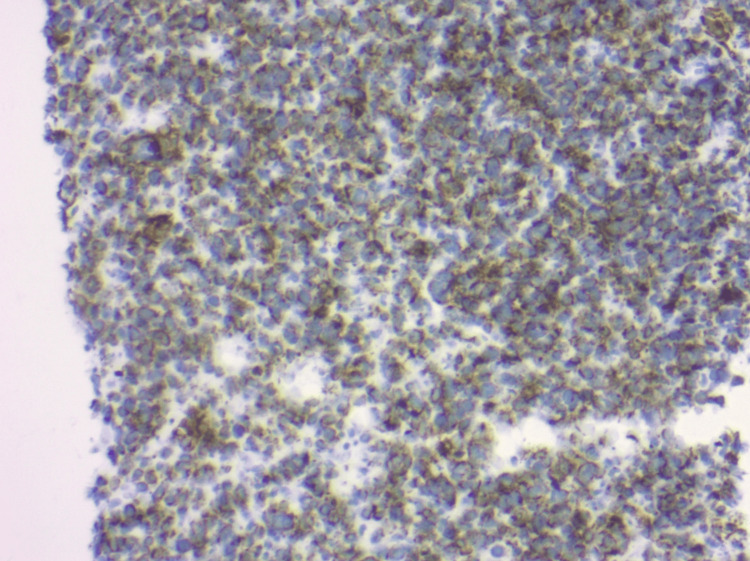
Liver biopsy (100x) showing positive pankeratin staining consistent with the diagnosis of small cell carcinoma

**Figure 6:**
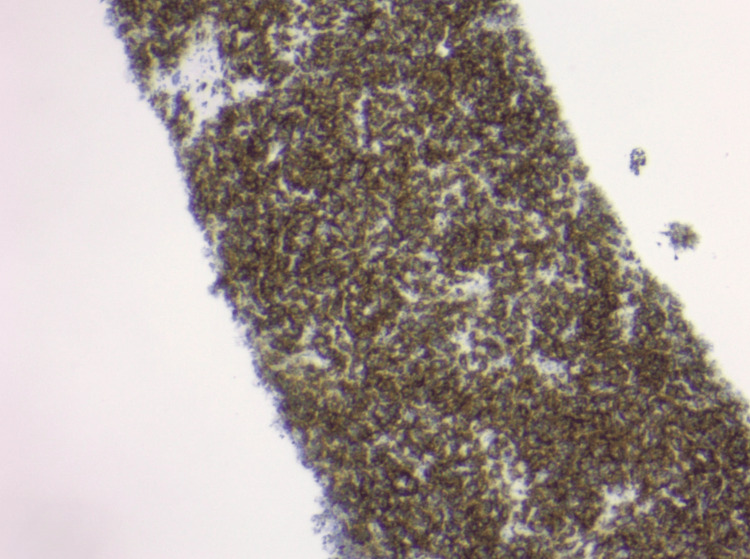
Liver biopsy (40×) demonstrating diffuse synaptophysin positivity, supporting neuroendocrine differentiation consistent with primary hepatic small cell carcinoma

The patient underwent a sinister clinical course. He developed grade 1 hepatic encephalopathy and progressive liver failure. He was treated with lactulose, rifaximin, and N-acetylcysteine.

A repeat CT of the abdomen and pelvis demonstrated a markedly enlarged liver measuring 24 cm with heterogeneous texture and scattered hyperdensities, most prominent in the right dome, with an infiltrative lesion measuring 5.5 × 3.5 cm. The portal vein, hepatic veins, and hepatic artery remained patent. No mass was identified in the lower lung fields. Trace perihepatic ascites was present.

Despite supportive care, he developed worsening liver failure, hypoxic respiratory failure, and shock. After transitioning to do-not-resuscitate/do-not-intubate status, he died seven days after admission from multisystem organ failure.

The diagnosis was favored to represent primary hepatic small cell carcinoma rather than metastatic pulmonary small cell carcinoma because chest radiograph and CT showed no lung mass or pulmonary malignancy, lower lung fields on abdominal CT were negative for mass, and the tumor was TTF-1 negative. In the setting of diffuse hepatic involvement, absence of an identifiable pulmonary primary, and positivity for a specific tumor marker, the findings supported a primary hepatic origin.

## Discussion

Extrapulmonary small cell carcinoma was first described by Duguid and Kennedy in 1930 and is recognized as a distinct entity from pulmonary small cell carcinoma. Establishing a primary extrapulmonary origin requires exclusion of a pulmonary primary through appropriate chest radiograph and clinical evaluation, as demonstrated in our patient, who had a negative chest radiograph and CT. The absence of a pulmonary lesion despite extensive hepatic involvement and the tumor’s immunohistochemical profile supported a primary hepatic origin rather than metastatic pulmonary disease [1].

We identified thirty-one additional cases of primary hepatic small cell carcinoma involving the liver reported across multiple individual case reports and small series [2-18]. Cases were identified through searches of Google Scholar, PubMed, ClinicalKey, the Cochrane Database, and Web of Science using the terms “primary hepatic small cell carcinoma,” “small cell carcinoma of the liver,” and related keywords. Only cases in which a pulmonary primary had been excluded were included. Shastri et al. performed a pooled analysis of reported cases and similarly highlighted the rarity of this malignancy and the limited data available regarding its clinical behavior and management [14]. Among 25 cases reporting sex, 68% were male, and the average age at diagnosis was 57 years (range 28-89 years). Our patient falls within the typical reported demographic range. Cases have been described across Asia, Europe, North America, and India, demonstrating the rarity but global distribution of this entity [2-18].

Among 18 cases with available medical history, 30% had active hepatitis or cirrhosis, and 28% had diabetes, findings that are notable given our patient’s suspected alcohol-related cirrhosis and diabetes mellitus [13-15]. Smoking history was documented in eight cases, with 63% reported as current or former smokers [10,13,16,17]. Although these associations have been observed repeatedly, the small number of reported cases does not permit definitive conclusions about risk factors. Chronic hepatic injury may contribute to malignant transformation through prolonged inflammation and cellular regeneration, potentially explaining the association between chronic liver disease and primary hepatic small cell carcinoma.

Abdominal pain, jaundice, and weight loss were the most frequently reported presenting symptoms. Physical examination commonly revealed abdominal tenderness, distension, scleral icterus, hepatosplenomegaly, or a palpable mass [8-11,16-18]. Our patient similarly presented with abdominal pain and was noted to have scleral icterus, jaundice, abdominal distension, and hepatosplenomegaly. Laboratory findings in prior reports frequently demonstrated hyperbilirubinemia and transaminitis, whereas alpha-fetoprotein and carcinoembryonic antigen were often within normal limits [2,4,9,10,14]. Consistent with these findings, our patient had marked hyperbilirubinemia and elevated transaminases despite a normal alpha-fetoprotein level. The absence of elevated tumor markers supports the possibility that malignancy should remain on the differential diagnosis despite normal tumor marker levels.

Imaging in previously reported cases most often demonstrated a dominant right hepatic mass, multifocal hepatic lesions, or diffuse hepatic involvement [4,8,11,13,16-18]. No cases described an isolated left-sided lesion. In our patient, imaging demonstrated diffuse hepatic involvement with an infiltrative appearance, consistent with patterns reported in the literature. However, a particularly notable feature of our case was the absence of hepatic lesions on the CT performed three months earlier. This rapid radiographic progression likely reflects the highly aggressive nature of small cell carcinoma and may account for the fulminant hepatic failure observed during hospitalization. Immunohistochemical staining in reported cases most commonly demonstrated positivity for CD56, synaptophysin, chromogranin, or neuron-specific enolase [2,4,8,14]. In our case, tumor cells were positive for CD56 and synaptophysin, supporting the diagnosis of small cell carcinoma.

Treatment was reported in 23 cases, with eight patients alive after intervention. Five survivors underwent combined surgical resection and platinum-based chemotherapy, most commonly cisplatin or carboplatin with etoposide [7,8,14]. Two patients survived with chemotherapy alone, and one patient survived following surgery alone with multiple resections over 32 years [8,12,17]. These findings suggest that combined modality therapy may offer the greatest opportunity for prolonged survival in selected patients with localized disease and preserved hepatic function. In contrast, our patient experienced rapid clinical deterioration and died seven days after admission, before oncologic therapy could be initiated. The diffuse hepatic involvement and advanced liver failure at presentation likely limited therapeutic options and contributed to the poor outcome.

A prior series of 34 extrapulmonary small cell carcinomas involving sites such as the esophagus, thymus, and stomach similarly reported the use of platinum-based chemotherapy regimens. Survival depended largely on tumor location and extent of disease, with worse outcomes observed in gastrointestinal sites and in patients with extensive disease burden [1]. The rapid progression in our patient is therefore consistent with the poor outcomes reported in gastrointestinal extrapulmonary small cell carcinoma.

Our review is limited by the small number of reported cases and inconsistent reporting of clinical variables. Publication bias may favor unusual or survivable cases, and the retrospective nature of available reports limits generalizability. Still, this case presents several important clinical lessons. Earlier consideration of infiltrative hepatic malignancy, prompt tissue diagnosis, and expedited multidisciplinary oncologic evaluation may improve treatment opportunities for future patients. Although the aggressive disease course in our patient may not have been reversible, earlier diagnosis before the onset of fulminant liver failure might have allowed consideration of systemic chemotherapy or combined modality treatment, which has been associated with the longest reported survivals in the literature [7,8,14]. This case illustrates the aggressive behavior of primary small cell carcinoma of the liver and demonstrates that rapid hepatic failure may occur.

## Conclusions

Primary small cell carcinoma of the liver is an exceptionally rare and highly aggressive malignancy that can present with rapidly progressive liver failure and nonspecific clinical findings. This case shows the importance of considering infiltrative hepatic malignancy in patients with acute liver dysfunction, particularly when tumor markers are unrevealing and recent imaging is negative. Prompt recognition, histopathologic confirmation, and exclusion of a pulmonary primary are essential, although outcomes remain poor because of the disease's aggressive nature.
